# Association between work-related musculoskeletal symptoms and quality of life among dental students: a cross-sectional study

**DOI:** 10.1186/s12891-022-04998-3

**Published:** 2022-01-10

**Authors:** Berkant Sezer, Sinan Kartal, Duygu Sıddıkoğlu, Betül Kargül

**Affiliations:** 1 Department of Pediatric Dentistry, School of Dentistry, Çanakkale Onsekiz Mart University, Cumhuriyet Mah. Sahilyolu Cd. No:5, 17100 Kepez, Merkez / Çanakkale Turkey; 2 Department of Restorative Dentistry, School of Dentistry, İzmir Katip Çelebi University, İzmir, Turkey; 3 Department of Biostatistics, School of Medicine, Çanakkale Onsekiz Mart University, Çanakkale, Turkey; 4 Department of Pediatric Dentistry, School of Dentistry, Marmara University, İstanbul, Turkey

**Keywords:** Musculoskeletal symptoms, Quality of life, Dental students

## Abstract

**Background:**

Dental students are frequently affected by work-related musculoskeletal symptoms (WMSs) due to reasons such as working conditions, difficult education process and long work periods. The aim of the study was to investigate the frequency and anatomical distribution of WMSs, and its effect on the quality of life (QoL) in dental students.

**Methods:**

Sociodemographic and health-related characteristics of one-hundred and five dental students were recorded. WMSs were scored by the participants with the Nordic Musculoskeletal Questionnaire. Then, participants were asked to evaluate their QoL by scoring the World Health Organization Quality of Life-Brief Form. Differences between independent groups for continuous variables were evaluated by Student’s t-test and ANOVA as appropriate. Linear regression analysis was performed to determine the effect of demographic and health-related parameters in predicting the QoL subscales.

**Results:**

The most common painful region in the last 12 months was the neck (66.7%). The body region with the most WMSs in the last 7 days was the upper back (43.8%). Physical health-related QoL of those with diagnosed musculoskeletal symptoms, and general health-related QoL of those using medicine due to any musculoskeletal symptoms were found to be statistically significantly lower (*p* = 0.018, *p* = 0.041, respectively). It was observed that the general and physical health, psychological well-being, and social relationship of the participants who reported the presence of neck pain in the last 7 days were statistically significantly lower (*p* = 0.003, *p* < 0.001, *p* = 0.004, *p* = 0.012; respectively). According to multiple regression analyses, pain occurrence in the body in the last 12 months and/or in the last 7 days had a negative impact on the participants’ general and physical health, psychological well-being, social relationship, and environmental status and related QoL (*p* = 0.026, *p* = 0.047, *p* = 0.021, *p* = 0.001, *p* = 0.027, respectively).

**Conclusions:**

The results of this study show that dental students’ body regions, especially the neck and the back, are affected by WMSs. These negative changes observed in the body had a negative effect on the QoL of the dental students.

## Background

Musculoskeletal diseases are characterized as work-related musculoskeletal diseases if they occur due to occupational factors such as inadequate and uncomfortable working conditions, long working hours, increased workload, and the position of the body during working periods [1]. Work-related musculoskeletal symptoms (WMSs) such as pain and incapacity frequently affect healthcare professionals worldwide. In particular, general dentists are considered to be one of the high-risk groups in this regard due to reasons such as long treatment sessions, continous force applied to the hand-wrist region, long-term static and/or wrong body position of the clinician, use of vibrating instruments and psychomotor skills [2, 3]. A meta-analysis [4] shows that the prevalence of WMSs in dental professionals varies between 10.8 and 97.9% in different countries. In addition, the most frequently affected body region is the neck, followed by the lower back, shoulders and the upper back [4]. Dental students performing clinical procedures simulate the work behaviour of dental professionals. Moreover, unlike licensed dentists, dental students perform clinical procedures without dental assistants. It is observed that a dental student frequently moves in various positions in a routine treatment process to reach different parts of the dental unit, such as the unit table, reflector, saliva ejector, and various materials within the clinic. Some of these movements are performed while standing and some are sitting on the dental stool. In addition, the fact that students have less coping skills compared to professionals and the presence of various psychosocial stress factors specific to dental school increase the prevalence of WMSs in dental students [5]. In 2021, Hashim et al. [6] showed that 48.5% of dental students had experienced musculoskeletal pain in the last week and 68.3% in the last year.

Quality of life (QoL) is a structure that affects individuals’ overall life satisfaction, emotional well-being, and emotional functioning. Although the concept of QoL does not have a universal definition because it is multidimensional, multicultural and complex, it can be explained as individuals’ perception of their position in life within the context of their culture, value systems, their goals, expectations, standards, and concerns regarding this situation [7, 8]. Systematic reviews show that dental students experience higher levels of stress than the general population and are more prone to burnout, anxiety, and depression [9, 10]. The long duration and demanding nature of education in dental schools can cause dental students to experience significant stress over extended periods [10]. In addition, WMSs may cause physical, social, psychological, and/or environmental consequences that negatively affect the lives of individuals [11]. Many studies show that WMSs observed in different healthcare workers disrupt QoL to varying degrees [12–16], and also that various social problems can occur due to reasons such as decreasing productivity and workforce [17]. However, to the best of our knowledge, there is no study evaluating the relationship between WMSs and QoL on dental students in the current scientific literature.

Therefore, the aim of the study was to investigate the frequency and anatomical distribution of WMSs, and its effect on the QoL in senior dental students.

## Methods

### Study design

Ethical approval for this cross-sectional and questionnaire-based study was approved by the Marmara University Institute of Health Sciences Ethics Committee with protocol number 261 in 2017. The study was carried out within all the ethical principles of the Declaration of Helsinki of 1975, as revised in 2008 [18].

The study was carried out with senior dental students of School of Dentistry, Marmara University that met the inclusion criteria. In Turkey, where dentistry education lasts for 5 years and ten semesters, the term “senior dental student” defines students studying in the 5th grade. Senior dental students have the ability to perform relatively long and invasive procedures of dentistry in addition to their 4th year clinical experience. Senior dental students formed the sample group of the study because they are more likely to suffer from WMSs because they perform longer and invasive clinical procedures without a dental assistant.

The questionnaire application and the measurement of body mass index (BMI) were carried out by one trained clinician.

### Participant recruitment

Upon giving the information to the study group about the research verbally, the informed-consent forms in which the same information was written were also distributed, and the participants were signed with their own sentences indicating that they have read and understood and voluntarily accepted to participate. The inclusion criteria of the study were: being a senior student at School of Dentistry, Marmara University, voluntarily accepting to participate in the study, filling out the questionnaire completely and correctly, not having a congenital and/or acquired disability, not having a previous severe trauma history, and not being pregnant for female participants.

### Questionnaire study

#### General information and measurement of body mass index

The first part of the questionnaire form includes various demographic and health-related questions asking age, gender, handedness, any diagnosed systemic disease, the education level of the mother and father, any diagnosed musculoskeletal disease, any medication usage due to musculoskeletal symptoms, and regular physical activity habits. Following the completion of the first part of the questionnaire, the clinician measured the participant’s weight and height using a standardized Health-O-Meter (HY-RGZ160 Weight & Height Measuring Scale, China). BMI (kg/m^2^) was measured with the index of weight adjusted for height square [19]. Then, depending on the result of this measurement, the clinician noted the participant as underweight, normal, or overweight. For most adults, an ideal BMI is in the 18.5 to 24.9 range. Individuals with a BMI score below 18.5 are considered underweight, individuals between twenty-five and 29.9 are considered overweight, and individuals over thirty are considered obese [19]. As none of the participants’ BMI scores in this study was above thirty, the BMI scores were divided into three groups.

#### Assessment of musculoskeletal symptoms

In the second part of the questionnaire form, the participants filled in the Nordic Musculoskeletal Questionnaire (NMQ) in order to evaluate the WMSs [20]. The NMQ was used in order to collect data about WMSs including pain and incapacity status during the last 12 months and 7 days in one of nine regions of the body (neck, shoulders, elbows, wrists and hands, upper back, lower back, hips and tights, knees, ankles and feet) and it was scored in twenty-seven questions with yes/no options [20]. The pain occurence reported by the participants in the last 12 months and 7 days is important because senior dental students have moved to a clinically more intensive grade in the last year and they perform more invasive and long-lasting clinical procedures in this process. The validity and the reliability study of NMQ in Turkish was performed by Kahraman et al. [21], and has appropriate psychometric properties, including good test-retest reliability, internal consistency, and construct validity.

#### Measurement of quality of life

In the last part of the questionnaire form, the participants filled in the World Health Organization Quality of Life-Brief Form (WHOQOL-BREF) in order to evaluate the QoL [22]. WHOQOL-BREF has been developed as a shortened version of WHOQOL-100; it can be used in situations where there are time constraints, when the burden of respondent needs to be minimized, or when some detailed parameters are unnecessary (such as in epidemiological studies and sometimes clinical studies). The WHOQOL-BREF consists of a total of 26 questions in four subscales (physical, psychological, social, and environmental) that evaluate QoL [22]. Since each subscale expresses the QoL in its subscale independent of each other, subscale scores are calculated between four and twenty. An increase in score means an increase in QoL. The scores obtained in this way are raw scores and the score is applied to the percentage system through its formula. In the physical health subscale, seven items were scored for pain and discomfort, use of medication, energy and fatigue, mobility, sleep and rest, routine daily activities, and working capacity; in the psychological subscale, six items scoring positive and negative emotions, spirituality, thinking, learning, memory, concentration, body appearance, and self-esteem; in the social relations subscale, there are three items in which personel relationships, sexual activity, and social support are scored; and eight items scoring physical safety and environment, financial resources, knowledge and skills, areas and skills to spend time, home environment, access to health and social care services, and transportation in the environment subscale. In addition, the questionnaire contains two items by which general health is scored [22]. The validity and reliability study of the WHOQOL-BREF in Turkish was performed by Eser et al. [23], and as a result of this study, it was stated that the Turkish version of the WHOQOL-BREF was acceptable, indicating that the scale is reliable and valid for healthcare workers.

### Statistical analysis

Categorical variables were expressed as frequencies and percentages (%) whereas continuous variables were expressed as mean ± standard deviation (SD) and as median (interquartile range – IQR) for the non-normally distributed variables. The Shapiro–Wilk test was used to assess the normality assumption for the continuous variables. Differences between independent groups for continuous variables were evaluated by Student’s t-test and ANOVA as appropriate. The significance of the difference between the groups in terms of the median values was analyzed using the Mann-Whitney U test. Controlling for demographic characteristics, regression models of the health-related explanatory parameters were explored with univariate and multivariable linear regression to determine possible independent predictors incorporating overall QoL and its subscales. Upon completion of the univariate analysis selected variables were adopted for the multivariate analysis. Studies show that the most affected body region of dental professionals by WMSs is the neck [4]. For this reason, the neck was evaluated as a separate body region, by clustering different regions in a group in order to make the statistical evaluation more expressive. In this context, the body is divided into four main regions: the neck, upper extremity, trunk, and lower extremity. The upper extremity includes shoulders, elbows, wrists and hands; the trunk includes upper and lower back; and the lower extremity includes hips and tights, knees, ankles and feet. Body regions were grouped in this way in order to obtain statistically interpretable results for linear regression equations and evaluating the effects symptoms seen in different body regions on QoL. The presence of pain in the last 12 months and/or last 7 days was considered as ‘pain occurence’ for regression models. All statistical analyses were conducted using SPSS 19.0 for Windows Version 19.0 software (IBM Corp., Armonk, NY, USA) and *p*-values of less than 0.05 were considered to indicate statistical significance.

## Results

### Description of participants

A total of 112 senior students studying at the School of Dentistry, Marmara University were invited to the study. Four students did not accept to participate in the study and three students filled in the questionnaires incompletely and/or incorrectly. Finally, the data of a total of 105 senior students who participated in this cross-sectional study were evaluated. Most of the participants in the study were female (65.7%). The participants are aged between twenty-two and thirty-four years (mean ± SD: 23.15 ± 1.70). Table 1 shows the various demographic and health-related data of the sample population.

**Table 1:** Sociodemographic and health-related characteristics of the participants

	N (%)
**Gender**	
*Female*	69 (65.7)
*Male*	36 (34.3)
**Body Mass Index (BMI)**	
*Underweight*	12 (11.4)
*Normal*	80 (76.2)
*Overweight*	13 (12.4)
**Handedness**	
*Left-handed*	9 (8.6)
*Right-handed*	96 (91.4)
**Presence a diagnosed systemic health problem**	
*Yes*	8 (7.6)
*No*	97 (92.4)
**Mother’s educational status**	
*Primary school*	28 (26.7)
*High school*	28 (26.7)
*University*	49 (46.7)
**Father’s educational status**	
*Primary school*	12 (11.4)
*High school*	35 (33.3)
*University*	58 (55.2)
**Presence of a diagnosed musculoskeletal disorder (except disabilities)**	
*Yes*	12 (11.4)
*No*	93 (88.6)
**Using medicine due to any musculoskeletal symptom**	
*Yes*	29 (27.6)
*No*	76 (72.4)
**Regular physical sports acitivity**	
*Yes*	49 (46.7)
*No*	56 (53.3)

*N* Number

### Distribution of work-related musculoskeletal symptoms

When the data of NMQ items were examined, it was seen that the most painful body region in the last 12 months was the neck (66.7%). In the last 12 months, incapacity to work due to pain was mostly seen in the lower back (19%). In the last 7 days, pain occurence was mostly seen in the upper back (43.8%). Table 2 shows the prevalence of pain and incapacity for each of the nine body regions of NMQ.

**Table 2:** Distribution and frequency of symptoms among body regions regarding the Nordic Musculoskeletal Questionnaire

Nordic Musculoskeletal Questionnaire items	Frequency of WMSs N (%)								
	***Neck***	***Shoulders***	***Elbows***	***Wrists and hands***	***Upper back***	***Lower back***	***Hips and Tights***	***Knees***	***Ankles and Feet***
***Pain occurrence in the last 12 months***	70 (66.7)	59 (56.2)	4 (3.8)	30 (28.6)	68 (64.8)	63 (60)	14 (13.3)	28 (26.7)	23 (21.9)
***Incapacity to work due to pain in the last 12 months***	14 (13.3)	13 (12.4)	1 (0.9)	6 (5.7)	17 (16.2)	20 (19)	2 (1.9)	9 (8.6)	6 (5.7)
***Pain occurrence in the last 7 days***	42 (40)	30 (28.6)	2 (1.9)	10 (9.5)	46 (43.8)	34 (32.4)	1 (0.9)	7 (6.7)	12 (11.4)

*WMSs* Work-related Musculoskeletal Symptoms, *N* Number

### Associations of work-related musculoskeletal symptoms and quality of life

The participants’ QoL and subscale scores are shown in Table 3. There was no significant relationship between gender, BMI, handedness, presence a diagnosed systemic health problem, and mother’s educational status and QoL scores (*p* > 0.05). General QoL score significantly differs regarding father education level (*p* = 0.006). Generally, participants whose father's education was at university level had higher mean QoL scores in all subscales. Participants who are using medicine due to any musculoskeletal symptoms have lower QoL scores as expected. It has been observed that environmental well-being is statistically different besides the education level of the father (*p* = 0.002). Physical health was significantly higher in participants who did regular sports activities and had not been diagnosed with any musculoskeletal disorder (*p* = 0.041, *p* = 0.018, respectively).

**Table 3:** The participants’ quality of life and subscale scores

	QoL General Health	QoL Physical Health	QoL Psychological	QoL Social Relationship	QoL Environment
***Participants’ (N = 105) scores (mean ± SD)***	61.1 ± 17.44	66.51 ± 12.92	62.1 ± 13.11	64.55 ± 15.41	60.64 ± 10.84

*QoL* Quality of Life, *N* Number, *SD* Standard deviation

When the relationship between WMSs including pain occurence, incapacity, and QoL was observed, incapacity to work due to neck pain in the last 12 months was statistically associated with lower scores on physical health and the social relationship subscales (*p* = 0.022, *p* = 0.009, respectively). In addition, the presence of pain in the neck in the last 7 days significantly decreased QoL in all subscales except the environmental effects (*p* < 0.05). It seems that the pain occurrence in the upper extremity in the last 12 months significantly decreased the individuals’ psychological well-being and related QoL (*p* = 0.024), incapacity to work due to pain in the last 12 months significantly decreased the physical health (*p* = 0.007), and the social relationship (*p* = 0.004), and the pain occurrence in the last 7 days significantly decreased the physical health (*p* = 0.037), and psychological well-being and related QoL (*p* = 0.015). It has been found that the pain occurrence in the trunk in the last 12 months has significantly decreased QoL in all subscales (*p* < 0.05). In addition, it has been observed that incapacity to work due to pain in the last 12 months significantly decreased physical health (*p* = 0.012), and the pain occurrence in the last 7 days significantly decreased physical health, psychological and environmental status and related QoL (*p* = 0.012, *p* = 0.001, *p* = 0.026, respectively). When the data of the lower extremity, which have the least impact on the QoL of senior dental students, are examined, it was observed that the incapacity to work due to pain in the last 12 months and the pain occurrence in the last 7 days significantly decreased the psychological well-being and related QoL (*p* = 0.008, *p* = 0.015, respectively).

### Effects of work-related musculoskeletal symptoms on quality of life

Table 4 summarized the effects of independent exploratory parameters on predicting overall QoL and its subscales. When the effects of various demographic and health-related factors on QoL and subscales in senior dental students are evaluated, it was observed that gender and BMI did not have a statistically significant effect on QoL (*p* > 0.05). For the general health-related QoL, fathers’ high educational status has a positive effect, whereas using medicine due to any musculoskeletal symptom and pain occurrence in the trunk has a negative effect on the general health-related QoL, the model was significant (F = 2.380, adjusted R^2^ = 0.298; *p* = 0.001, *p* = 0.038, *p* = 0.026, respectively). For the physical health-related QoL, while presence of a diagnosed musculoskeletal disorder and pain occurrence in the trunk effects negatively, regular physical sports activity has a positive effect on the physical health-related QoL, the model was significant (F = 3.622, adjusted R^2^ = 0.233; *p* = 0.017, *p* = 0.017, *p* = 0.047, respectively). For the psychological status-related QoL, using medicine due to any musculoskeletal symptom and pain occurrence in the trunk controlled, the model was signficant (F = 5.957, adjusted R^2^ = 0.427; *p* = 0.008, *p* = 0.021, respectively;). For the social relationship-related QoL, only pain occurrence in the trunk showed a significantly negative effects with this subscale (adjusted R^2^ = 0.349, β = − 0,471, *p* = 0.001). For the environmental status-related QoL, fathers’ educational status, and pain occurrence in the trunk controlled, the model was significant (F = 6.229, adjusted R^2^ = 0.244; *p* = 0.025, *p* = 0.027, respectively).

**Table 4:** Summary of multiple linear regression predicting the quality of life and its subscales

	QoL General Health			QoL Physical Health			QoL Psychological			QoL Social Relationship			QoL Environment		
	***B***	***β***	***p-value***	***B***	***β***	***p-value***	***B***	***β***	***p-value***	***B***	***β***	***p-value***	***B***	***β***	***p-value***
**Constant**	90.968		0.001	90.376		< 0.001	92.805		< 0.001	79.829		0.001	81.853		< 0.001
**Age**	−1.253	−0.123	0.223	−0.821	−0.108	0.302	− 0.845	− 0.108	0.296	− 0.508	− 0.060	0.572	− 0.902	− 0.142	0.178
**Gender (reference: Male)**	0.704	0.019	0.865	1.834	0.065	0.568	−4.411	−0.152	0.178	2.267	0.071	0.539	−0.025	−0.001	0.993
**BMI (reference: Normal)**															
**Underweight**	−5.073	−0.100	0.511	−4.290	−0.114	0.473	2.602	0.067	0.668	0.736	0.018	0.914	2.260	0.072	0.652
**Overweight**	7.491	−0.106	0.370	12.182	−0.232	0.062	5.371	−0.099	0.414	0.734	−0.012	0.921	−1.739	0.039	0.749
**Mother’s educational status (reference: Primary school)**															
**High school**	−5.814	−0.125	0.288	−1.708	−0.050	0.686	−4.518	−0.127	0.294	−5.528	−0.142	0.275	−4.306	−0.149	0.228
**University**	−6.834	−0.164	0.291	1.935	0.063	0.698	1.559	0.049	0.759	1.533	0.044	0.786	1.963	0.076	0.640
**Father’s educational status (reference: Primary school)**															
**High school**	−9.450	−0.176	0.106	4.041	0.102	0.370	8.241	0.201	0.074	0.105	0.002	0.984	1.763	0.053	0.641
**University**	22.772	0.456	**0.001**	1.843	0.050	0.712	−0.669	−0.017	0.895	4.723	0.114	0.405	9.528	0.307	**0.025**
**Presence of a diagnosed musculoskeletal disorder**				−10.024	−0.253	**0.017**									
**Using medicine due to any musculoskeletal symptom**	−8.546	−0.219	**0.038**				8.719	0.292	**0.008**						
**Regular physical sports acitivity**				6.426	0.249	**0.017**									
**Pain occurrence in the trunk**	−12.533	−0.283	**0.026**	−8.635	−0.264	**0.047**	−10.202	−0.302	**0.021**	−17.273	−0.471	**0.001**	−8.102	−0.295	**0.027**
**R**^**2**^	0.298			0.402			0.427			0.349			0.244		
**F**	2.380		0.007	3.622		0.005	5.957		0.001	6.639		0.002	6.229		0.014

*QoL* Quality of Life, *BMI* Body-Mass Index, *B* unstandardized regression coefficient, *β* standardized regression coefficient. Bold values mean statistically significant *(p < 0.05)*

## Discussion

Studies show that WMSs are frequently seen in the practice of the dental profession with the effect of work-related and other environmental factors [24, 25]. The reasons why WMSs are seen more frequently in dental students are the difficulty of the education and training process, intensive theoretical and practical training and exams, and being inexperienced compared to a dental professional [26]. Since it is very important to manage WMSs well and to determine the factors in order for individuals to fulfill their duties effectively, it is also necessary to consider this situation in dental students. WMSs are a highly influential factor not only on work and service continuity, but also on individuals’ QoL. Many studies have shown the decrease in QoL due to WMSs in different business lines and/or societies [12, 14, 27]. Although there are studies in the scientific literature that examine the presence, frequency, and distribution of WMSs in dental students, there is no existing study evaluating the effects of WMSs on dental students’ QoL. From this point of view, this study is a first and its results should be evaluated carefully.

Pain occurrence and incapacity to work affect different body regions in different occupational groups and time intervals. In different studies conducted on dental students and dental professionals, it has been stated that the most affected body region due to WMSs is the neck [4, 25]. In parallel with these findings, according to the results of this study, the most affected region of the body in the last 12 months was the neck. On the other hand, the upper back and lower back are other regions that are frequently affected by those who practice dentistry [25]. According to the results of this study, the lower back was the body region where incapacity to work due to pain was observed most frequently in the last 12 months. In the last 7 days, it was observed that the most common pain occurrence was in the upper back, neck, and lower back, respectively. In this context, it can be said that, according to NMQ data, the most frequently affected body regions of senior dental students are the neck, lower back, and upper back. In the study conducted by Ohlendorf et al. [25], it was found that the most frequently affected body regions were the neck, lower back, and upper back in dentists and dental students, in parallel with this study’s findings. According to the results of a meta-analysis [4] that compiled the results of forty-one studies conducted in Western countries, the most affected body region is the neck, followed by the back. In the study of Botta et al. [28], in which dental students evaluated the risk factors for musculoskeletal disorders, as a result of the NMQ evaluation, the first body region affected in terms of the pain occurrence in the last 12 months was the neck with 73.79%, followed by the lower back with 62.06%. According to the results of the research conducted by Khan and Chew [29] on the prevalence of musculoskeletal disorders in clinical and non-clinical dental students, the most frequently affected body region in clinical dental students was the neck; it was followed by the upper back and lower back. Although the ranking was the same in non-clinical students, its prevalence was lower. This situation can be explained by the physical movements performed during the practice of dentistry, the occurrence of psychological factors as a result of the patient-physician relationship, and the more difficult practical training in higher classes [5, 25]. In a study by Tezel et al. [30] in which a group of dental students were investigated for the presence of musculoskeletal disorders according to their handedness status, neck pain was the most common in the whole study group, followed by shoulder pain and back pain. The results of a systematic review of WMSs in dental professionals showed that the most frequently observed WMSs were in the back and then in the neck [31]. As observed in the aforementioned studies and the results of this study, the reasons for the frequent pain occurrence and incapacity to work in dental students in the upper part of the body such as the neck, upper back, and shoulders are muscle fatigue due to abnormal static postures that compress blood vessels and reduce oxygenation during patient care [32, 33]. The pain occurrence in the lower back is related to factors that cause muscle imbalance, such as repetitive unilateral bending, insufficient lumbar support for long work periods, and non-ergonomic dental stool design [28]. Although the results of observational studies conducted with dental professionals and dental students in different countries and the reviews along with meta-analyses conducted at different times show that the most frequently affected body regions by WMSs are the neck, back, and shoulders in general, the reasons for the change in ranking are the clinical intensity of physicians in different countries and times, ergonomic working conditions and knowledge, and the presence of other external determinants that may affect WMSs.

When WMSs are evaluated on the QoL, it has been observed that the presence of a diagnosed musculoskeletal disorder has a significant effect on the physical health of individuals; it has been observed that using medicine due to any musculoskeletal symptom has a significant effect on general health, and according to the multiple linear regression results, it has an effect on psychological well-being; and it has been observed that regular physical sports activity has a significant effect on physical health as expectedly. It is known that chronic musculoskeletal pain causes long-term activity limitations in individuals [34]. There is also a strong relationship between pain and reduced physical activity. The reduction in physical activity also negatively affects overall physical health, associated with a progressive decrease in muscle strength and flexibility. The severity, duration, and region of the pain plays a critical role in individuals’ physical health [35, 36]. In addition, over time, chronic pain and incapacity to work, and consequently using medication, trigger fear and anxiety related to pain. This causes depression, which further decreases daily function and QoL with activity avoidance [34]. This situation may explain the decrease in the psychological subscale scores. Although pain occurrence and incapacity to work due to pain in the last 12 months and pain occurrence in the last 7 days in different regions of the body affected different QoL subscales at different levels, multiple linear regression results showed that the pain observed in the trunk, including the upper back and lower back, significantly increased all subscale scores of QoL. In the study of Antonopoulou et al. [37], it was observed that especially upper back and neck pain had a significant negative effect on the QoL subscale scores. The results of Koyuncu and Karcioglu’s research [38], in which healthcare professionals in internal medicine, general surgery, and emergency departments examined the effect of musculoskeletal complaints on quality of work life, showed that the presence of musculoskeletal complaints, especially lower back pain and symptoms, had a negative effect on quality of work life. Deduced from the results of many studies mentioned, there are negative effects of WMSs on QoL in different professions.

One of the main limitations of this study is that the sample size is relatively small, since the study was conducted with senior dental students only. The data of students who received preclinical education or who have not yet received practical training may change the current results. In addition, the fact that the study was conducted at a single state university dental school is another limitation. Findings may vary as public and private universities in different cities of the country have different environmental and educational factors. This study was conducted before the COVID-19 pandemic. During the pandemic period in Turkey, it has experienced troubles in practical and clinical dental training. For this reason, it will be useful to repeat the study during and after the pandemic period. Finally, there is a possibility that the WMSs and QoL parameters may change under different factors, since not all participants have the same facilities. Only some sociodemographic and health-related determinants were examined in our study. The effects of more and different sociodemographic and sociocultural factors on WMSs and related QoL can be evaluated.

## Conclusions

Dentistry is a profession where certain physical and mental variables are at the prefront, and these variables affect the QoL of dentists. In addition to these factors in dental students, difficulties brought by clinical intensity and inexperience may further exacerbate WMSs. The results of this study show that dental students’ body regions, especially the neck and the back, are affected by WMSs. These negative changes observed in the body had a negative effect on the QoL of the dental students. In this context, it is important that dental schools organize their education in a way that is of higher quality that causes less physical and mental fatigue in order to prevent students from losing their belief in and motivation for the profession. In addition, providing ergonomic working conditions and relevant training, along with having feedback on WMSs, will further improve QoL.

## Data Availability

The datasets used and/or analyzed during the current study are available from the corresponding author on reasonable request.

## Abbreviations

BMI: Body Mass Index
Corp.: Corporation
COVID-19: Coronavirus disease-2019
IBM: International Business Machines
IQR: Interquartile range
kg: Kilogram
m^2^: Square meters
N: Number
NMQ: Nordic Musculoskeletal Questionnaire
NY: New York
QoL: Quality of life
SD: Standard deviation
SPSS: Statistical Package for the Social Sciences
USA: United States of America
WHOQOL-BREF: World Health Organization Quality of Life-Brief Form
WMSs: Work-related musculoskeletal symptoms

## References

[1] Violante FS (2020). Criteria for diagnosis and attribution of an occupational musculoskeletal disease. Med Lav.

[2] Petromilli Nordi P, Polli GS, Campos JA (2013). Working postures of dental students: ergonomic analysis using the Ovako working analysis system and rapid upper limb assessment. Med Lav.

[3] Vijay S, Ide M (2016). Musculoskeletal neck and back pain in undergraduate dental students at a UK dental school - a cross-sectional study. Br Dent J.

[4] Lietz J, Kozak A, Nienhaus A (2018). Prevalence and occupational risk factors of musculoskeletal diseases and pain among dental professionals in Western countries: a systematic literature review and meta-analysis. PLoS One.

[5] Thornton LJ, Barr AE, Stuart-Buttle C (2008). Perceived musculoskeletal symptoms among dental students in the clinic work environment. Ergonomics..

[6] Hashim R, Salah A, Mayahi F, Haidary S (2021). Prevalence of postural musculoskeletal symptoms among dental students in United Arab Emirates. BMC Musculoskelet Disord.

[7] Garzaro G, Clari M, Donato F (2020). A contribution to the validation of the Italian version of the work-related quality of life scale. Med Lav.

[8] Andre A, Pierre GC, McAndrew M (2017). Quality of life among dental students: a survey study. J Dent Educ.

[9] Alzahem AM, van der Molen HT, Alaujan AH, Schmidt HG, Zamakhshary MH (2011). Stress amongst dental students: a systematic review. Eur J Dent Educ.

[10] Elani HW, Allison PJ, Kumar RA, Mancini L, Lambrou A, Bedos C (2014). A systematic review of stress in dental students. J Dent Educ.

[11] Luque-Suarez A, Martinez-Calderon J, Falla D (2019). Role of kinesiophobia on pain, disability and quality of life in people suffering from chronic musculoskeletal pain: a systematic review. Br J Sports Med.

[12] Bae YH, Min KS (2016). Associations between work-related musculoskeletal disorders, quality of life, and workplace stress in physical therapists. Ind Health.

[13] Ribeiro T, Serranheira F, Loureiro H (2017). Work related musculoskeletal disorders in primary health care nurses. Appl Nurs Res.

[14] Yan P, Yang Y, Zhang L (2018). Correlation analysis between work-related musculoskeletal disorders and the nursing practice environment, quality of life, and social support in the nursing professionals. Medicine (Baltimore).

[15] Serra MVGB, Camargo PR, Zaia JE, Tonello MGM, Quemelo PRV (2018). Effects of physical exercise on musculoskeletal disorders, stress and quality of life in workers. Int J Occup Saf Ergon.

[16] Giagio S, Volpe G, Pillastrini P, Gasparre G, Frizziero A, Squizzato F (2019). A preventive program for work-related musculoskeletal disorders among surgeons: outcomes of a randomized controlled clinical trial. Ann Surg.

[17] Karakaya İÇ, Karakaya MG, Tunç E, Kıhtır M (2015). Musculoskeletal problems and quality of life of elementary school teachers. Int J Occup Saf Ergon.

[18] World Medical Association Declaration of Helsinki, Ethical Principles for Medical Research involving human subjects. https://www.wma.net/wp-content/uploads/2018/07/DoH-Oct2008.pdf. Accessed 11 Oct 2021.

[19] Centers for Disease Control and Prevention, Healthy Living, About Adult Body Mass Index. https://www.cdc.gov/healthyweight/assessing/bmi/adult_bmi/index.html. Accessed 11 Oct 2021.

[20] Kuorinka I, Jonsson B, Kilbom A (1987). Standardised Nordic questionnaires for the analysis of musculoskeletal symptoms. Appl Ergon.

[21] Kahraman T, Genç A, Göz E (2016). The Nordic musculoskeletal questionnaire: cross-cultural adaptation into Turkish assessing its psychometric properties. Disabil Rehabil.

[22] Skevington SM, Lotfy M, O'Connell KA, WHOQOL Group (2004). The World Health Organization's WHOQOL-BREF quality of life assessment: psychometric properties and results of the international field trial. A report from the WHOQOL group. Qual Life Res.

[23] Eser E, Fidaner H, Fidaner C, Eser SY, Elbi H, Göker E (1999). Psychometric properties of the WHOQOL-100 and WHOQOL-BREF. J Psychiatry Psychol Psychopharmacol.

[24] Koni A, Kufersin M, Ronchese F, Travan M, Cadenaro M, Larese FF (2018). Approach to prevention of musculoskeletal symptoms in dental students: an interventional study. Med Lav.

[25] Ohlendorf D, Naser A, Haas Y (2020). Prevalence of musculoskeletal disorders among dentists and dental students in Germany. Int J Environ Res Public Health.

[26] Faust AM, Ahmed SN, Johnston LB, Harmon JB (2021). Teaching methodologies for improving dental students' implementation of ergonomic operator and patient positioning. J Dent Educ.

[27] Dick RB, Lowe BD, Lu ML, Krieg EF (2020). Trends in work-related musculoskeletal disorders from the 2002 to 2014 general social survey, quality of work life supplement. J Occup Environ Med.

[28] Botta AC, Presoto CD, Wajngarten D, Campos JADB, Garcia PPNS (2018). Perception of dental students on risk factors of musculoskeletal disorders. Eur J Dent Educ.

[29] Khan SA, Chew KY (2013). Effect of working characteristics and taught ergonomics on the prevalence of musculoskeletal disorders amongst dental students. BMC Musculoskelet Disord.

[30] Tezel A, Kavrut F, Tezel A, Kara C, Demir T, Kavrut R (2005). Musculoskeletal disorders in left- and right-handed Turkish dental students. Int J Neurosci.

[31] Hayes M, Cockrell D, Smith DR (2009). A systematic review of musculoskeletal disorders among dental professionals. Int J Dent Hyg.

[32] Oliveira Dantas FF, de Lima KC (2015). The relationship between physical load and musculoskeletal complaints among Brazilian dentists. Appl Ergon.

[33] Park HS, Kim J, Roh HL, Namkoong S (2015). Analysis of the risk factors of musculoskeletal disease among dentists induced by work posture. J Phys Ther Sci.

[34] Tüzün EH (2007). Quality of life in chronic musculoskeletal pain. Best Pract Res Clin Rheumatol.

[35] Ang DC, Kroenke K, McHorney CA (2006). Impact of pain severity and location on health-related quality of life. Rheumatol Int.

[36] Kayo AH, Peccin MS, Sanches CM, Trevisani VF (2012). Effectiveness of physical activity in reducing pain in patients with fibromyalgia: a blinded randomized clinical trial. Rheumatol Int.

[37] Antonopoulou MD, Alegakis AK, Hadjipavlou AG, Lionis CD (2009). Studying the association between musculoskeletal disorders, quality of life and mental health. A primary care pilot study in rural Crete, Greece. BMC Musculoskelet Disord.

[38] Koyuncu N, Karcioglu Ö (2018). Musculoskeletal complaints in healthcare personnel in hospital: an interdepartmental, cross-sectional comparison. Medicine (Baltimore).

